# The effect of salt water ageing on the mechanical and rheological properties of magnetorheological elastomer

**DOI:** 10.1038/s41598-023-33171-6

**Published:** 2023-04-10

**Authors:** Muntaz Hana Ahmad Khairi, Saiful Amri Mazlan, Ubaidillah Ubaidillah, Rahayu Emilia Mohamed Khaidir, Nur Azmah Nordin, Mohd Aidy Faizal Johari, Siti Aishah Abdul Aziz, Salihah Tan Shilan, Seung-Bok Choi

**Affiliations:** 1 Engineering Materials & Structures (eMast) ikhoza, Malaysia-Japan International Institute of Technology (MJIIT), Universiti Teknologi Malaysia, 54100 Kuala Lumpur, Malaysia; 2 Mechanical Engineering Department, Faculty of Engineering, Universitas Sebelas Maret, J1. Ir. Sutami 36A, Ketingan, Surakarta, 57126 Central Java Indonesia; 3 Faculty of Applied Sciences, Universiti Teknologi MARA Pahang, 26400 Bandar Tun Abdul Razak Jengka, Malaysia; 4 Pusat Asasi Stem, Universiti Malaysia Terengganu, 21030 Kuala Nerus, Malaysia; 5 Department of Mechanical Engineering, The State University of New York, Korea (SUNY Korea), 119 Songdo Moonhwa-Ro, Yeonsu-Gu Incheon, Incheon, 21985 Republic of Korea

**Keywords:** Engineering, Materials science

## Abstract

This paper aims to investigate the mechanical and rheological properties of magnetorheological elastomer (MRE) in marine ecosystems. The prepared samples comprised silicone rubber (SR) and 70 wt% micron-sized carbonyl iron particles (CIPs), immersed in an artificial marine ecosystem using salt water (Natrium Chloride) for 30 days. The mechanical properties of MRE samples were evaluated using hardness and quasi-static tensile tests. While the rheometer was used to investigate the rheological properties of their storage modulus condition with magnetic field stimulation. Further analysis of the defects and damages caused by salt water ageing was done through morphological observation using scanning electron microscope (SEM) technology. The results showed that the hardness and tensile strength of MRE samples that were soaked in salt water were affected over time. Lower values of hardness and tensile strength were obtained after 30 days due to the presence of Na^+^ and Cl^−^, which acted as an accelerator to the hydrolyzation process of the MRE. The process then, enhanced the water ingress capability into the matrix to cause the molecular changes. Interestingly, for rheological properties, 30 days of salt water ageing allowed the water molecules to move the MRE matrix molecular chains apart, a process known as plasticization and thus increasing the MR effect. Furthermore, morphological evidence was established to determine the MRE changes during salt water ageing. The research findings should greatly contribute to a better understanding of the effect of salt water on the performance of MRE.

## Introduction

The advancement of smart material technology and application has diverged into many service environments including aquatic ecosystems, covering marine and freshwater. As the marine environment comprised 70% globally, the potential of smart materials being used in these surroundings is very promising. Currently, conventional rubber-based materials are widely used in many marine-related applications, particularly for isolating the unwanted vibration of offshore structures caused by wave or earthquake load, this catastrophic leads to a reduction in service life and fatigue failure of the offshore platforms^1,2^. With the limitation faced by these conventional rubber materials, there is a need for replacing them with rubber-based smart materials that can prolong the performance of the product. Besides, these materials can provide real-time controllability of stiffness and damping as semiactive devices for mitigating the excessive vibrations of platform structure induced by wave, wind and earthquake loads.

Offshore platform design specification requires schedule replacement, especially for the rubber part, which is known to suffer from salt contamination and fatigue loading from weather hazard that expedites the process of ageing^3^. As a result, understanding the behavior, reaction and gradual ageing process of smart materials is important, especially to be utilized in isolator devices. Besides, these materials have been engineered to change some of their characteristics in response to external stimuli^4,5^. Magnetorheological elastomer (MRE) is one of the smart materials that can alter its rheological properties reversibly upon the application of a magnetic field^6–10^. Furthermore, MRE is a contender for the role of the material that can be used in offshore platforms for extended performance.

Several phenomena can happen in aquatic ecosystems when a rubber/polymeric-based material is immersed in water through physical and chemical processes. Firstly, according to Lettieri and Frigione^11^, the physical process was involved when the water molecules move polymer chains apart (free volume). This phenomenon is also known as plasticization, which leads to a decrease in the stiffness of the polymer. Secondly, according to Azwa et al.^12^, water absorbed in polymers was either free water or bound water that indicated the chemical processes. Water molecules that are relatively free to travel through the microvoids and pores are identified as free water, while those dispersed in the polymer matrix and attached to the polar groups of the polymer are designated as bound water^13^. Eventually, the excessive absorption of water then decreases the free water and simultaneously increases the bound water^14^. The phenomenon leads the water molecules from bound water to cut the polymer chains by scission via hydrolysis and degraded the polymers. Where, hydrolysis is a chemical process of decomposition involving the splitting of a matrix molecular bond and the addition of the hydrogen cation and the hydroxide anion of water.

On the other hand, polymer hydrolysis is happened more rapidly in marine water (hereafter referred to as salt water) than in fresh water. This is proven by the analysis done by the different authors on the impact of salt water on the physical properties of polymeric-based materials^3,12,14–21^. The studies implied that Na^+^ and Cl^−^ ions in salt water accelerate hydrolyzation by increasing the ability of water to diffuse more through the matrix. As a result, the polymer breaks down faster in salt water than in fresh water. In addition, the degree of degradation has more significantly changed with a longer aging period^8^ In another study, Wang et al.^20^ revealed polymerics that contain metal part more worsen as results in a decreased interfacial adhesion of polymer/metal bonding. Therefore, MRE can be considered as a polymeric material that contains metal parts (CIPs) that may have a similar issue.

As a potential material to be used in marine industries, MRE needs to undergo a salt water ageing test to understand the ability of MRE to withstand and perform well during unfavourable conditions. Up to now, the mechanical and rheology properties of MRE under salt water ageing are not been explored. Thus, the purpose of this work is to investigate the initial effect of salt water on the performance of MRE by immersing the MRE sample for 30 days in salt water with 35% of salinity. The findings will enable manufacturing industries to design MRE components that are significant in marine industries, thereby extending the device’s lifetime.

## Results and discussion

### Hardness tests

The resistance of a material to permanent indentation is referred to as its hardness measured by a spring-loaded needle-like indenter. Table 1 shows the measured hardness of MRE and SR samples before and after the salt water immersion.

**Table 1:** Hardness measurement for MRE samples.

No	Sample name	Shore-A
1	MRE-0d	60
2	MRE-30d	51
3	SR-0d	50
4	SR-30d	42

Due to the 70 wt% CIPs embedded in the rubber matrix, the MRE-0d produced the highest hardness. The finding was in line with the previous report, which claimed that the hardness increased with the addition of metal particles to the polymer matrix^22^. After 30 days of the salt water immersion, even though sample MRE-30d contained the same 70 wt% CIPs, the hardness was decreased by 15% to 51 shores A. The subsided has come from the matrix, where the water molecule has altered the molecular chain structures of MRE. The water molecules, which behaved as a plasticizer in the matrix, then contributed to an increase in molecular chain mobility. This action disrupted chain entanglements and cohesive forces between the molecular chains. As a result, the matrix became soft hence lowering the hardness of MRE-30d. Similarly, the hardness value for SR-30d has reduced by 16% as compared to SR-0d, which was higher than MRE. The decrease was due to no embedded CIPs in the SR that restricted molecular chain mobility.

### Morphology

**Figure 1:**
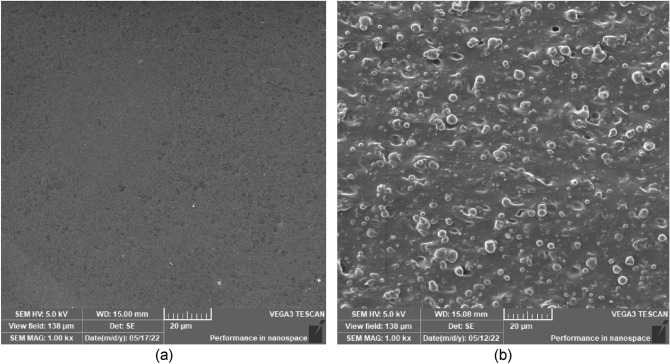
SEM micrographs for (**a**) surface morphology and (**b**) cross-section of MRE sample.

**Figure 2:**
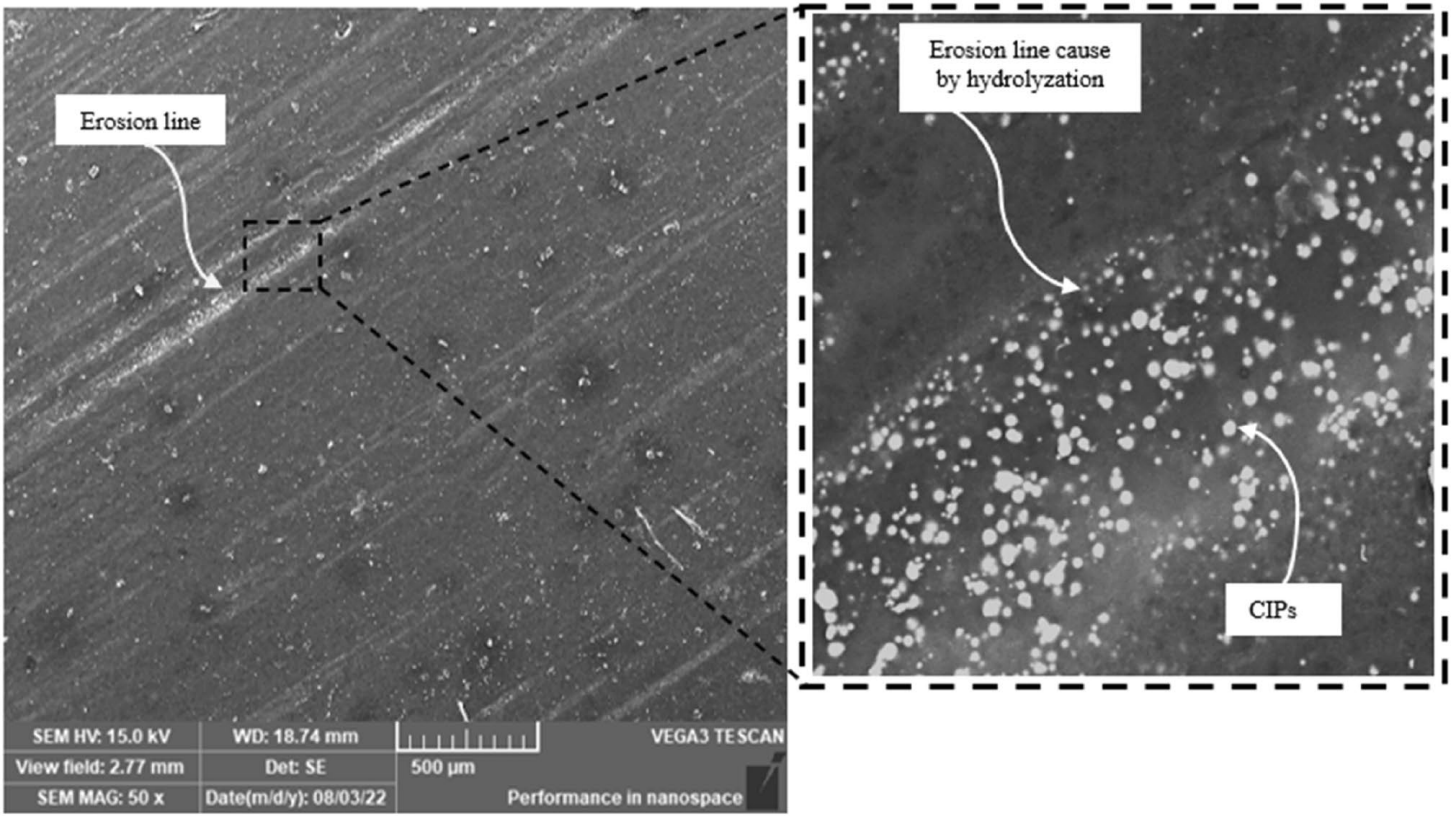
Surface morphology of MRE samples after 30 days of immersion in salt water with magnification picture inside the small box.

Fabricated samples were morphologically analysed to look for any flaws or deficiencies that might occur during the fabrication process. Surface and cross-section micrographs of MRE samples are shown in Fig. 1. LV-SEM analysis of the MRE surface reveals a smooth surface and embedded CIPs in the matrix of cross-sectioned MRE are shown in Fig. 1a and b, respectively. The micrograph displayed that CIPs were uniformly distributed in the MRE in which demonstrating the nature of the isotropic sample. Nonetheless, after 30 days of immersion in salt water, the MRE sample developed a surface defect, as depicted in Fig. 2. Immersion in salt water changed the property of MRE and generated micro defects on its surface, where erosion lines appeared on the surface. The same phenomenon was also found by Johari et al.^23^ by exposing MRE to a natural weathering environment in tropical climates. The micro-sized eroded lines on the matrix surface exhibited CIP’s surface, which for this finding was the initiation of degradation by hydrolyzation. During the salt water immersion, ions in the salt solution and water infiltrated into the matrix, diffused and changed the backbone structures of molecular chains^14,21^. Thus, it was crucial to comprehend the deterioration of the MRE surface due to submersion in salt water, as it was correlated with the expected service life of the MRE.

### Mechanical properties

After 30 days of immersion in salt water solution, the mechanical properties of MRE were evaluated using a uniaxial tensile machine. To comprehend the impact of salt water on the matrix itself, the test was also conducted on an SR sample. MRE in this study consists of 30 wt% part of the matrix that contributed to its less cross-linked amorphous structure, therefore, limiting their elasticity. In contrast, SR which was occupied by a 100% matrix possesses a great cross-linked amorphous structure. In the case of MRE, despite having a low elongation due to its less amorphous structure, it has a crystallinity structure that contributed from CIPs, which represented its strength. MRE and SR samples demonstrated their natural behaviour following the unfilled and filled elastomer’s rule before salt water ageing test.

**Figure 3:**
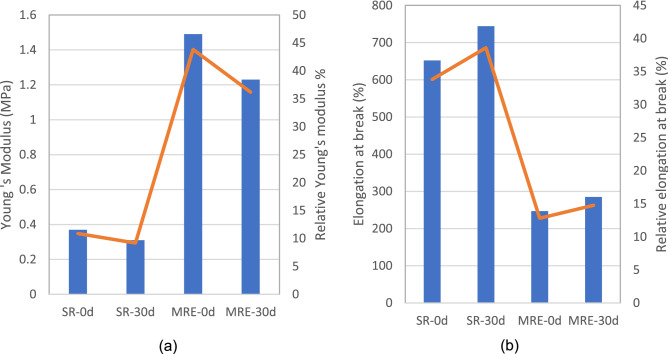
MRE and SR samples before and after immersion in the salt water (**a**) Young’s modulus and (**b**) elongation at break.

Figure 3 depicts Young’s modulus and elongation at break for both MRE and SR samples before and after the immersion in salt water. Both MRE and SR samples after 30 days of immersion in salt water showed a decrease in elasticity and further the strength of the samples. As for the MRE samples, the result showed that Young’s modulus measured for the MRE-30d of salt water ageing was 1.23 MPa as compared to 1.49 MPa for MRE-0d. The finding indicated that the modulus value was decreased by 17%. Similarly, Young’s modulus value for SR-30d was reduced by 16% as compared to SR-0d. Unavoidably meant that the material experienced changes even in just 30 days, most likely as a result of a disturbance in the rubber molecular chains by the salt water. In the salt water environment, water molecules penetrated the matrix through micro-cracks by hydrolyzation and reduced interfacial adhesion of CIPs with the matrix^13^. Moreover, Cl^−^, Na^+^ ions increased water diffusion ability in the matrix due to water acting as a plasticizer and permitting the matrix molecular to move with each other that consequently softening the matrix system^13,14,21^. Therefore, the process of hydrolyzation and plasticization resulted in reducing Young’s modulus of MRE and SR.

As illustrated in Fig. 3b, the MRE-0d sample had an elongation of 247% before salt water ageing and increased by 15% after 30 days. Water frequently acted as a potent plasticizer and many amorphous solids absorbed water from their surroundings on their own^24^. Therefore, in this salt water ageing test, the plasticization not only affected Young’s modulus but affected the elongation as well. The water molecules facilitated the movement of the rubber molecular chains among themselves hence increasing the rubber chains’ mobility and thus increasing the sample's elongation. Meanwhile, the elongation at break for SR-0d was 652% which was about 2 times higher than the MRE-0d and increased by 14% for SR-30d. Amorphous molecular structures of the matrix were formed before the salt water ageing test with the initial cross-linkage arrangements generated during the solidification process with the platinum-based curing catalyst. The cross-linking was formed with a long and entangled amorphous structure that allowed it to be stretched to its maximum elongation. The SR sample exhibited the greatest elongation in its original state due to its high elasticity. The cross-linkages formed during the solidification process determined the elasticity and deformation limits of MRE.

### Rheology test- MR effect

**Figure 4:**
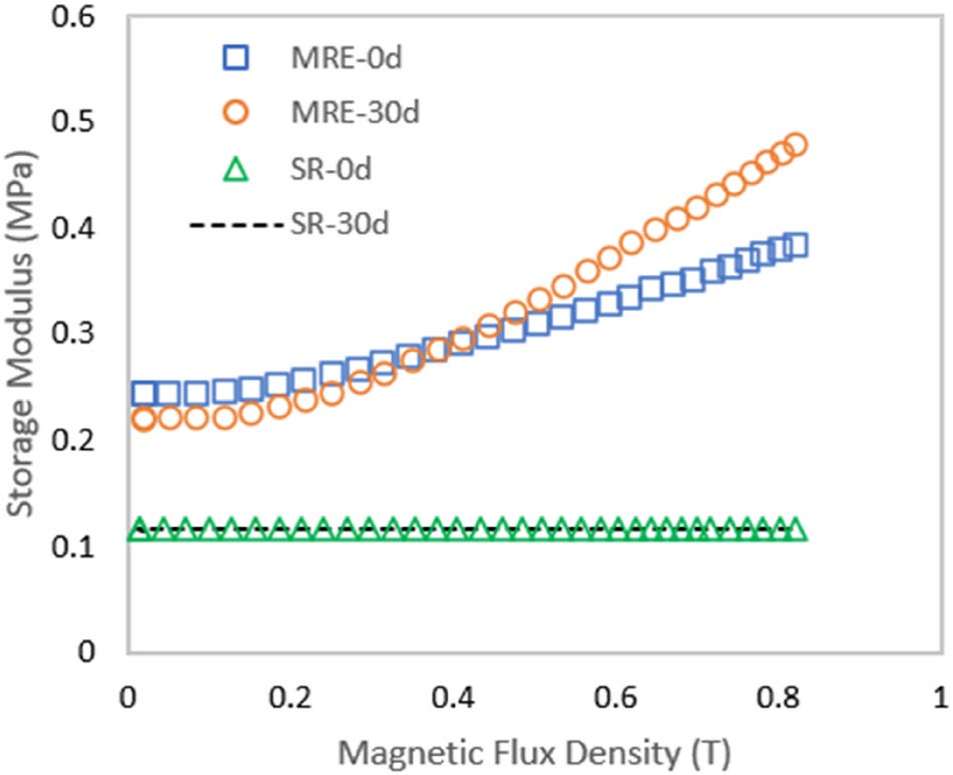
Storage modulus of SR and MRE samples versus magnetic flux density.

Figure 4 depicts the shear storage modulus (G’) of MRE at various magnetic flux densities. The storage modulus of all samples increased as the magnetic flux density increased from 0 to 800 mT. Even though all MRE samples had a fixed content of CIPs of 70 wt%, it was interesting to see how salt water immersion affected the curves by showing different increased patterns from the initial to the maximum value with the increment of the magnetic field density. In general, the initial storage modulus in the absence of a magnetic field was dependent on the modulus nature of the sample. Thus, the initial storage modulus of both MRE and SR and was slightly different in value. As shown in Fig. 4, MRE-0d and MRE-30d produced about an 8% difference in the initial modulus of 0.24 and 0.22 MPa, respectively. The reduction trend also occurred in SR sample where the initial storage modulus for SR-30d was reduced by 4% as compared to SR-0d. The changes in the molecular structure of both MRE-30d and SR-30d from the immersion of salt water reduced the stiffness of the sample, hence lowering the initial storage modulus.

Meanwhile, at on-state conditions, the stiffness (or modulus) of all MRE samples was changed due to the interaction of magnetic particles caused by the magnetic field application. Table 2 presents the rheological properties in terms of initial storage modulus *G*′_*0*_, maximum storage modulus *G*′_*max*_, magneto-induced modulus *∆G’*, MR effect, and relative MR effect of each sample.

**Table 2:** The rheological properties of SR and MRE samples.

Sample name	G′_0_ [MPa]	G′_max_ [MPa]	Magneto-induced, ∆G′ [MPa]	Relative MR effect (%)
MRE-0d	0.24	0.36	0.13	56.5
MRE-30d	0.22	0.48	0.26	118
SR-0d	0.12	0.12	–	–
SR-30d	0.11	0.11	–	–

The magneto-induced and relative MR effects of MRE-30d demonstrated higher values than MRE-0d. It was noted that no changes in the storage modulus for SR sample under the influence of the magnetic field due to no presence of CIPs. Thus, no value in both MR effects was recorded. Nonetheless, MRE-0d has a relative MR effect of 57% with 0.13 MPa magneto-induced modulus. Meanwhile, immersion of MRE-30d in salt water for 30 days has changed the molecular structure due to the penetration of the salt water into the MRE. During the immersion, water molecules moved the matrix molecular chains apart (plasticization process) so that, physically, the matrix became softer. Thus, this condition enhanced the interaction between CIPs as the matrix was less restricted when subject to the magnetic field. As a result, MRE-30d has the highest value of relative MR effect of 118% with magneto-induced modulus of 0.26 MPa at the maximum magnetic flux density of 0.8 T. Other than the plasticization effect, the MRE-30d also faced the early stage of hydrolysis. In salt water, Cl^−^, Na^+^ ions increased water diffusion ability in the matrix and accelerated hydrolyzation of the matrix molecular chains. Hydrolysis weakens the polymer since the backbone structure was altered^14,21^. However, the plasticization effect was dominant at this stage after 30 days of immersion as demonstrated by the elongation result in Fig. 3b in the previous sub-section, therefore it contributed to the enhancement of MR effect.

## Methods

### Preparation of MRE

In this study, an isotropic silicone-based MRE was used. The MRE samples consisted of silicone matrix embedded with 70 wt % carbonyl iron particles (CIPs), supplied by BASF OM, Germany, was fabricated using a closed mold. The CIPs were in a powder form and spherical shape with an average diameter of 6 µm. The CIPs were mechanically deposited and mixed into a silicone rubber, supplied by Nippon Steel Co., Japan, at a constant temperature of 25 °C. The mixtures were cured by adding a curing catalyst agent of platinum-based, NS625B (Nippon Steel) of 5 wt% in a square mold with the male and female parts. The curing process then took place, allowing uncured MRE to solidify after 2 h. The dimension of MRE sheet was 150 mm in length, 150 mm in width, and 1 mm in thickness, and it was cured in off-state conditions (no external magnetic stimuli). Excessive matrix bleed-out along the edges of the MRE sheet was removed with a trimming knife. A similar procedure was used to prepare the silicone rubber sheet (without CIP) when subjected to saltwater ageing as a control experiment. The MRE and silicone rubber samples were labelled as MRE-0d and SR-0d before salt water immersion and MRE-30d and SR-30d after 30 days of immersion, respectively.

### Salt water immersion procedure

**Figure 5:**
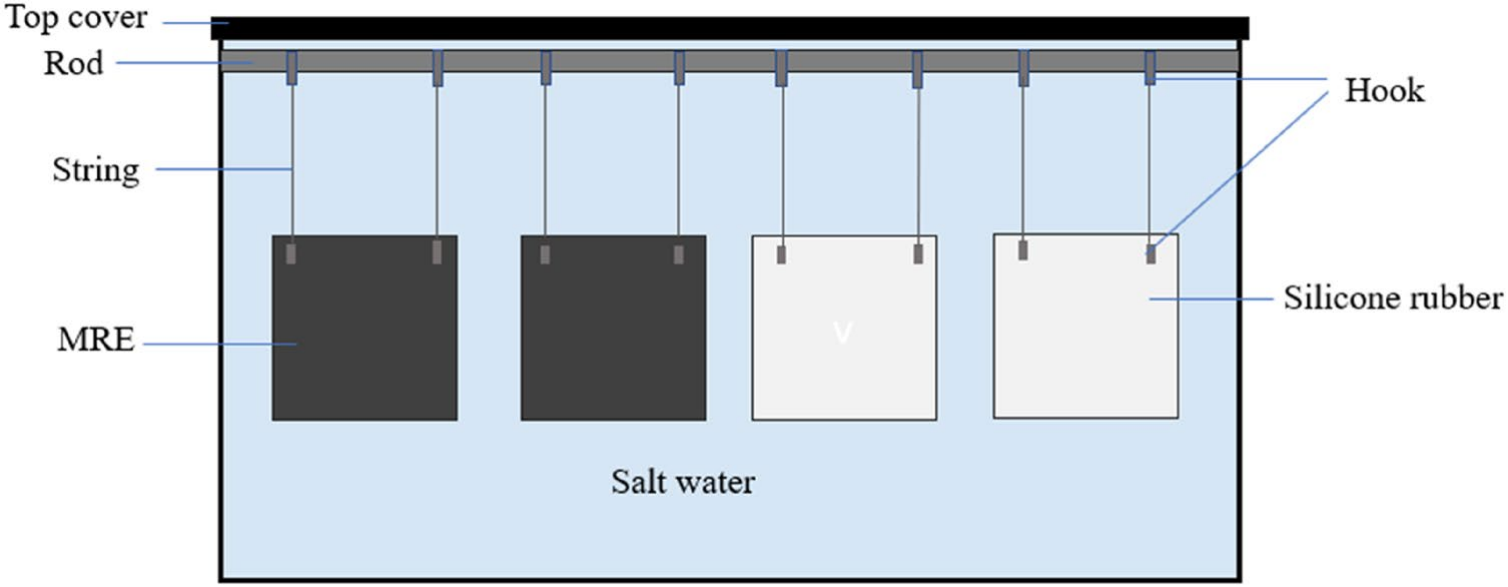
Arrangement of MRE and SR sheet in a salt water filled-container.

The procedure for salt water test was carried out by referring salt water salinity of 35% based on the global average salinity, which means that 1 kg of seawater contains 35 g of NaCl^18^. Salinity is the average mineralization of seawater that is used to express salt water concentration. In this study, salt water environment was prepared by measuring out the required amounts of tap water and sea salt (NaCl) supplied by R&M Chemicals (India). The NaCl was thoroughly mixed with room temperature tap water until completely dissolved for 10 min. The solution was stored in a glass container, and the measured pH level of the salt water was 8. Besides, as a reference to a similar matrix employed in the MRE, silicone rubber sheets (without CIPs) were prepared and immersed in salt water similarly. Then, the MRE and silicone rubber sheets were hung inside the salt water-filled container using a string in as shown in Fig. 5. The sheets were collected after 30 days of being immersed in salt water. Before progressing to the next step, the sheets were rested in the air for 24 h at room temperature.

### Hardness and microstructure analysis procedure

The hardness of the MRE samples was determined using a Durometer Shore A (Sauter HBA 100-0, Freiburg, Germany) according to ASTM D2240 standard. The test was performed five times on each sample to ensure that the results were consistent, and the average value was then calculated. Scanning electron microscopy (SEM) (Tescan Vega 3, Czech Republic) was used to investigate the physical aspects of the samples. Samples size of 1 mm × 10 mm, coated with a thin layer of gold at approximately 1 nm thickness using an auto fine sputter coater device (NS800, Novatic Scientific, Singapore) were prepared for microstructure analysis. Before the experimental evaluation, the primary focus was on the sample surface area before and after immersion in salt water. The surface was inspected for any flaws such as cracks or erosion. In addition, the cross-section of the initial sample was closely examined to determine the particle distribution within the matrix. Following the evaluation of mechanical properties, the fractured sample underwent similar procedures. The surfaces of the fractured samples were examined to determine the properties of the quasi-static tensile loading used after the test. The cross-section of the sample was observed in the gauge length area of both fractured parts of the sample.

### Mechanical test procedure

The dumbbell shapes of MRE samples were cut from the sheets using tensile cutting dies following the ASTM D412 test standard. The MRE sample was positioned parallel to the tensile load equipped with a load cell. The tensile strength and elongation at break of MRE samples were obtained using a universal testing machine, AG–X Series by Shimadzu, Kyoto, Japan with a crosshead speed of 500 mm/min at 25 °C according to the same test standard. An average of 5 samples was taken for each set of data.

### Rheological test procedure

A commercial rheometer was used to examine the field-dependent rheological properties of MRE samples (Physica MCR 302, Anton Paar, Graz, Austria). The rheometer is equipped with a controllable magnetic field, supported by MR device (MRD70/1 T) and profiled parallel plate measuring system (PP20/MRD/T1/P2). An MRE sheet was punched-cut to the desired diameter of 20 mm and nominal thickness of 1 mm using a hollow hole punch tool. A sample was placed between the equipment base and a rotating disc, and the test was performed in oscillatory motion in a shear mode at a constant temperature of 25 °C. The magnetic flux density values for each applied current were recorded automatically using a Teslameter connected to a rheometer. The values of magnetic flux density generated by setting the current to be applied are shown in Table 3.

**Table 3:** The relationship between applied current and magnetic flux density.

Applied current (A)	Magnetic flux density (T)
0	0.00
1	0.18
2	0.38
3	0.56
4	0.72
5	0.82

In this paper, the current sweep tests were used to investigate the rheological properties of MRE, particularly the MR effect. The frequency was set to 1 Hz at a constant strain amplitude of 0.01%. The relative MR effect was measured using Eq. (1) below.

1 $$\frac{{G}_{max}-{G}_{0}}{{G}_{0}} \times 100\%,$$

where *G*_*max*_ is the maximum storage modulus and *G*_*0*_ is the initial storage modulus.

## Conclusions

Understanding the ageing performance of MRE under seawater environments requires complex and critical studies of the water penetration processes into MRE, and the mechanism of degradation through physical and chemical processes. In this study, the mechanical and rheological properties of MRE were investigated after 30 days in salt water. The morphological observation revealed that surface deterioration by water hydrolysis and free volume was responsible for the development of the erosion and flaws lines. Thus, the results for hardness, Young’s modulus and elongation performance of MRE were decreased by 15, 17 and 15%, respectively, after 30 days of immersion in salt water. On the other hand, the rheology properties showed that the MR effect of MRE increased by 61.5% after 30 days of salt water ageing. The increase in the free volume due to the water molecules moving the matrix molecular chains apart of the MRE appeared as a plasticization effect that contributed to the result obtained. The findings should be noted as useful information for demonstrating the MRE’s resistance to salt water as well as a useful reference for future research in determining solutions to develop new MREs with better resistance to actual marine industries.

## Data Availability

The datasets used and/or analysed during the current study available from the corresponding author on reasonable request.
